# Unconventional slowing down of electronic recovery in photoexcited charge-ordered La_1/3_Sr_2/3_FeO_3_

**DOI:** 10.1038/s41467-018-04199-4

**Published:** 2018-05-04

**Authors:** Yi Zhu, Jason Hoffman, Clare E. Rowland, Hyowon Park, Donald A. Walko, John W. Freeland, Philip J. Ryan, Richard D. Schaller, Anand Bhattacharya, Haidan Wen

**Affiliations:** 1 Advanced Photon Source, Argonne National Laboratory, Argonne, IL 60439 USA; 2 Materials Science Division, Argonne National Laboratory, Argonne, IL 60439 USA; 3 Center for Nanoscale Materials, Argonne National Laboratory, Argonne, IL 60439 USA; 4 Department of Chemistry, Northwestern University, Evanston, IL 60208 USA; 5 Department of Physics, University of Illinois at Chicago, Chicago, IL 60607 USA; 6 School of Physical Sciences, Dublin City University, Dublin, 9 Ireland

## Abstract

The coupling of ordered electronic phases with lattice, spin, and orbital degrees of freedom are of central interest in strongly correlated systems. Their interplay has been intensively studied from femtosecond to picosecond time scales, while their dynamics beyond nanoseconds are usually assumed to follow lattice cooling. Here, we report an unusual slowing down of the recovery of an electronic phase across a first-order phase transition. Following optical excitation, the recovery time of both transient optical reflectivity and X-ray diffraction intensity from the charge-ordered superstructure in a La_1/3_Sr_2/3_FeO_3_ thin film increases by orders of magnitude as the sample temperature approaches the phase transition temperature. In this regime, the recovery time becomes much longer than the lattice cooling time. The combined experimental and theoretical investigation shows that the slowing down of electronic recovery corresponds to the pseudo-critical dynamics that originates from magnetic interactions close to a weakly first-order phase transition.

## Abstract (web summary)

Unusual electronic behavior can emerge in complex oxides due to strong coupling between charge, magnetic and lattice degrees of freedom. Zhu et al. observe separation of electronic and lattice equilibration times in La_1/3_Sr_2/3_FeO_3_ as magnetic interactions make the recovery of charge order much slower than lattice relaxation.

## Introduction

The interactions between electronic, spin, and structural degrees of freedom in correlated materials are the basis of emergent phenomena, including high-temperature superconductivity, metal-to-insulator phase transitions, and colossal magnetoresistance^1–3^. Strong correlations amongst these degrees of freedom hold promise for engineering material properties using targeted optical excitation to create hidden phases that do not exist in thermal equilibrium. These hidden phases can live as short as a few picoseconds, such as transient superconductivity in cuprates^4,5^, or can be long-lived metastable states, such as those in photoexcited manganites^6^ and dichalcogenides^7^. Understanding and possibly controlling how driven quantum systems achieve equilibrium is of central interest in nonequilibrium physics^8^ and for elucidating the lifetime of emergent photoinduced phenomena at hierarchical time scales.

The charge-ordered state, where patterns of charge density spontaneously emerge below a critical temperature *T*_c_, is one of the most interesting collective electronic phases and plays a critical role in determining the properties of many correlated materials^9^. Thanks to the distinct dynamics associated with different interaction mechanisms, complex interactions amongst multiple degrees of freedom, such as charge ordering (CO), lattice distortion, and spin ordering, can be effectively disentangled on ultrafast time scales, motivating studies of femtosecond-to-picosecond (fs-ps) responses^10–14^. Far below *T*_c_, CO can be quenched by ultrafast optical excitation and typically recovers on ps time scales, during which its relation with nonequilibrium structural distortions has been experimentally studied^15–18^. When the system temperature is close to *T*_***c***_, the initiation and recovery of photoinduced changes are drastically different from the case far below *T*_c_^10^. For example, a critical slowing down of charge density waves on ps time-scales was observed in cuprates^19^, molybdenum oxides^20,21^, and chalcogenides^11,22^. The recovery of charge ordering in layered organic salts can approach nanosecond (ns) time-scales due to electronic instabilities^23^. On longer time scales, after the charge, spin, and lattice degrees of freedom have had sufficient time to exchange energy to reach the same temperature, the evolution of electronic properties usually follows the cooling of the system through thermal exchange with the environment. Thermal recovery hereafter refers to the film lattice cooling due to the heat transport to the substrate. Although the glass-like recovery of antiferromagnetic spin order can be extremely slow, electronic recovery does not necessarily follow the recovery of spin order^24^. On long time-scales beyond those typically associated with thermal recovery, electronic slowing down has not been studied, and its microscopic mechanisms and the relation to other degrees of freedom are not clear. Understanding the role of correlations on these mesoscopic time-scales is essential to extending the lifetime of exotic electronic phases beyond thermal cooling of the system.

**Fig. 1:**
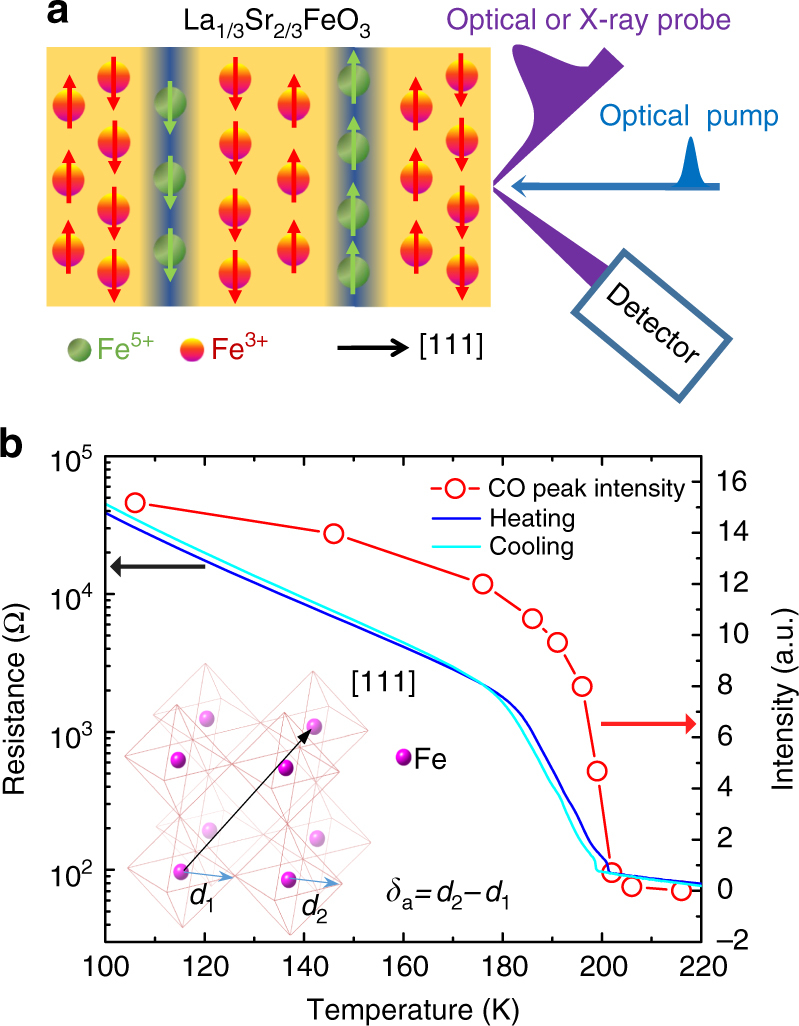
Experimental setup and static sample characterization. **a** Schematic drawing of the LSFO charge and AFM ordering and the time-resolved optical and X-ray diffraction experiments. Below *T*_c_, Fe^3+^, and Fe^5+^ ions order to form a periodic charge density distribution along the pseudo cubic [111] direction. Upon above-band-gap optical excitation, the transient optical reflectivity and the time-resolved X-ray diffraction from CO $\frac{4}{3}\frac{4}{3}\frac{4}{3}$ superlattice and 222 lattice were measured. **b** The temperature-dependent CO diffraction intensity (red circle) and the resistance (blue for heating and cyan for cooling) of the sample. The inset shows oxygen octahedra with the differentially averaged Fe–O length *δ*_a_ = *d*_2_*-d*_1_

Here, we report on an unusually slow recovery of a collective electronic phase that becomes significantly longer than the lattice cooling time near *T*_c_ in photoexcited La_1/3_Sr_2/3_FeO_3_ (LSFO) thin films. Using time-resolved optical spectroscopy and X-ray diffraction, we directly track three quantities in the time domain: the optical reflectivity, the superlattice X-ray diffraction peaks related to the order parameter of CO, and the lattice constant of LSFO (Fig. 1a). When the sample temperature increases towards *T*_c_, we observe concurrent slowing down of the recovery of both transient optical reflectivity and CO superlattice diffraction peak intensity, well beyond the few-ns thermal recovery of the independently characterized out-of-plane lattice constant . Thus, the time scale for lattice cooling does not determine the time scale of electronic recovery. Moreover, the X-ray diffraction measurements reveal important structural information: no significant change of the CO domain size is observed, which suggests a mesoscopic nucleation and growth scenario is not the dominant mechanism for the slowing down. By density functional theory plus *U* (DFT + *U*) calculations, we find that the magnetic-exchange-driven phase transition is weakly first order, in which phase separation of two competing CO configurations occurs close to *T*_c_. The recovery of CO following pathways along the temperature-dependent potential energy surfaces to the ground state qualitatively explains the observed slowdown. The scaling of the time constants as a function of temperature is ascribed to pseudo-critical dynamics in a weakly first-order transition^25^, driven by magnetic exchange interactions.

## Results

### Static sample characterization

The perovskite oxide La_1/3_Sr_2/3_FeO_3_ is a prototypical material in which the oxygen-mediated superexchange interaction between Fe sites drives a first-order metal-to-insulator (MIT) phase transition^26–28^. The MIT, paramagnetic to antiferromagnetic (AFM) transition, and CO transition occur concurrently at a transition temperature of around 200 K. Seventy nanometer LSFO thin film samples were grown by ozone-assisted molecular beam epitaxy on (111) SrTiO_3_ (STO) substrates. The *T*_c_ of the LSFO films was experimentally identified as the temperature at which charge ordering emerges (Fig. 1b). Upon cooling below *T*_c_, charge disproportionation between the Fe sites leads to ordered planes of Fe^5+^–Fe^3+^–Fe^3+^ stacked along the [111] direction in the pseudo-cubic representation^26^, accompanied by a sharp hysteretic rise in resistivity (Fig. 1b). While the reported valence states of Fe ions vary from 3+ to 5+ ^27,28^, an accompanying periodic structural distortion gives rise to CO superlattice (*n* ± 1/3, *n* ± 1/3, *n* ± 1/3; *n* is a positive integer) peaks in X-ray diffraction measurements (Supplementary Fig. 2)^28,29^. Unlike organic Mott insulators^23^, the formation of CO in LSFO is a first-order phase transition driven by magnetic interactions^26–28^.

### Transient electronic and structural dynamics

The transient electronic and structural dynamics were measured by time-resolved optical reflectivity and X-ray diffraction at the Center of Nanoscale Materials and the Advanced Photon Source at Argonne National Laboratory (see Methods). Calibrated sample temperatures are used throughout this paper (see Supplementary Note 3). In both optical and X-ray measurements, the photon energy of the pump laser is above the optical absorption edge of the LSFO thin film (2.2 eV)^30^. The penetration depth of the pump laser is comparable to the optical probing depth of 30 nm^30^, but less than the thickness of the film (70 nm). The mismatch between pump and probe penetration depth in the X-ray measurements is not a concern on the time scale of interest here (ns to μs), which is well beyond the time scale of the lattice thermalization within the film^31^.

**Fig. 2:**
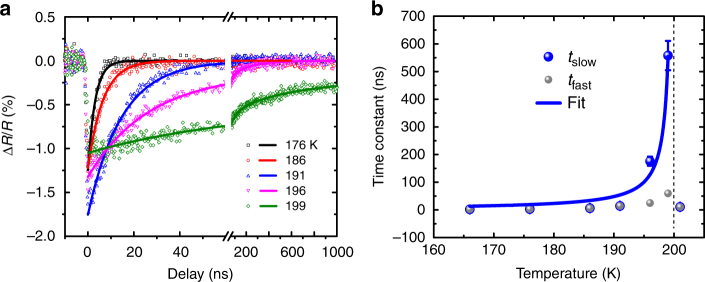
Transient optical reflectivity measurements. **a** Transient reflectivity changes probed at the wavelength of 1100 nm at various sample temperatures. Solid lines are fits with the fitting errors showing as error bars in **b**. **b** Recovery time constants of the transient reflectivity change as a function of sample temperatures. The solid line is a fit of *t*_slow_ to the scaling law $\sim (1 - T/T_{\mathrm c})^{ - \Delta }$

The changes of optical reflectivity measured at 1.1 μm following optical excitation with an absorbed pump fluence of 2.9 mJ cm^−2^ are shown at various temperatures in Fig. 2a. The recovery process can be fit by an exponential decay function ${\mathrm{\Delta }}R\left( t \right)\sim A_1\exp \left( { - {\textstyle{t \over {t_{{\mathrm{fast}}}}}}} \right) + A_2\exp \left( { - {\textstyle{t \over {t_{{\mathrm{slow}}}}}}} \right)$ with time constants *t*_fast_ and *t*_slow_. Far below *T*_c_, e.g., at *T* = 176 K, the fitting yields *t*_fast_ = *t*_slow_ ~2.3 ns, thus, a single exponential decay is sufficient to describe the recovery dynamics. However, Fig. 2b shows that, as the sample temperature approaches *T*_c_, *t*_slow_ increases by orders of magnitude, while *t*_fast_ does not change significantly. This observation indicates that *t*_slow_ is related to a non-trivial recovery mechanism, while *t*_fast_ is consistent with thermal recovery as discussed later. Measurements performed above *T*_c_ show a similar, several-ns recovery to that observed far below *T*_c_, suggesting the recovery of optical reflectivity above *T*_c_ is mainly driven by thermal recovery. The decrease in reflectivity at 1.1 μm upon optical excitation and the slowing down of the recovery are universal across the probed optical spectrum from 0.9 to 1.3 μm (Supplementary Note 1). The change of optical reflectivity is consistent with the spectral weight transfer of the optical conductivity from around 1 eV to low energy (<0.5 eV) as the film temperature increases^32^. This spectral range is ascribed to the transition from O 2*p* to Fe^3+^/Fe^5+^ *e*_*g*↑_ states and is closely related to the macroscopic Drude response across the metal-to-insulator phase transition^32^.

**Fig. 3:**
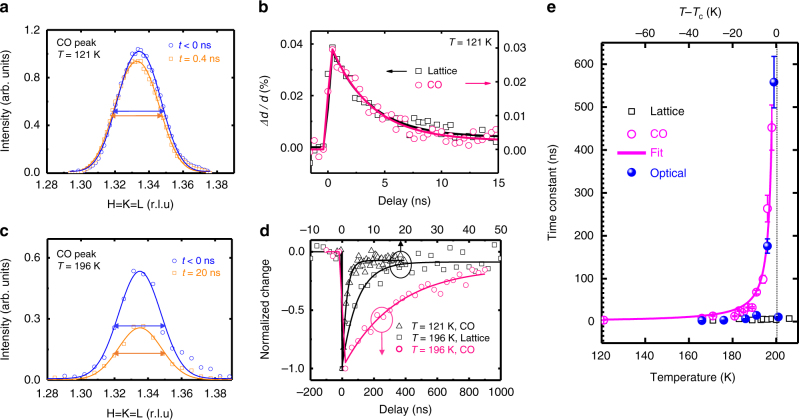
Time-resolved X-ray diffraction measurements. **a** Radial scans of CO peak measured at 121 K at various delays. The solid lines are Gaussian fits. The double-arrow lines with identical length show that the full width half maxima of the peaks before and after excitation are equal within the resolution. The HKL are indexed with respect to the LSFO ground-state lattice at specified temperatures. **b** Out-of-plane strain of lattice (black square) and CO superlattice (pink circle) as a function of the delay, with the fit result shown as solid lines. **c** Radial scans of CO peak measured at 196 K at various delays. **d** CO peak intensity as a function of delay is measured at *T* = 121 K (black triangles) and 196 K (pink circles). Lattice strain as a function of time measured at *T* = 196 K (black squares). **e** Recovery time constants of CO intensity τ_CO_ and lattice peak shift *τ*_lattice_ measured as a function of sample temperatures at an absorbed pump laser fluence of 2.9 mJ cm^−2^. The error bars show the fitting error. The solid line shows the fit of X-ray data based on the power law *(1−T/T*_c_*)*^*−Δ*^. The dotted line indicates the transition temperature. The blue solid balls show the recovery time constant of optical reflectivity, also shown in Fig. 2b

In order to understand the slowing down of the recovery of the electronic degree of freedom, we studied the recovery of CO-induced structural distortion, as well as that of the lattice constant, by monitoring the $\frac{4}{3}\frac{4}{3}\frac{4}{3}$ and 222 X-ray diffraction peaks, respectively. Because the lattice remains rhombohedral (R-3c space group) across *T*_c_, the time-dependent lattice constant provides an independent measurement of the film temperature as the lattice returns to thermal equilibrium. At *T* = 121 K, the CO recovery follows the lattice dynamics. Radial scans of both $\frac{4}{3}\frac{4}{3}\frac{4}{3}$ and 222 peaks were measured as a function of delay between the pump laser and probe X-ray pulses. The intensity of the superstructure peak decreases as CO melts, while the peak center shifts to lower HKL, indicating a superlattice expansion (Fig. 3a). The expansion strain of 0.04% measured by 222 peak at 100 ps corresponds to the film temperature increase of 24 K, calculated using the thermal expansion coefficient of 1.55 × 10^−5^ K^−1^ ^33^ and Poisson’s ratio of 0.32^34^ (See Supplementary Note 2). Figure 3b shows that the photoinduced strain measured by the shifts of the $\frac{4}{3}\frac{4}{3}\frac{4}{3}$ and 222 peaks relax at the same rate. The intensity of the superstructure peak (not shown) and transient expansion of the lattice can both be fit by a single exponential function with recovery time constants, *τ*_CO_ = 3.1 ns and *τ*_lattice_ = 2.8 ns, in good agreement with the recovery time constant of optical reflectivity at a temperature far from the CO phase transition. These observations show that, at temperatures far below *T*_c_, the recovery of the electronic degree of freedom is determined by the cooling rate of the thin film. Figure 3c shows the radial scans of the CO peak before and after laser excitation for a temperature closer to *T*_c_ (*T* = 196 K). The CO peak intensity is significantly suppressed upon laser excitation. Due to the mismatch between optical excitation depth and film thickness, the CO only partially melts and the measured diffraction peak is a sum of the residual unmelted CO and the recovering CO states. Nevertheless, no discernible change in the CO peak width was observed along the out-of-plane direction (Fig. 3c) or along the in-plane direction (Supplementary Fig. 8), indicating a nearly constant CO domain size during the recovery of CO state upon photo-excitation. Compared with the CO intensity recovery measured at 121 K in Fig. 3d, the recovery time of the CO peaks increases two orders of magnitude from 3.5 ns at 121 K to 263 ns at 196 K. Meanwhile, the recovery time of 222 peak shift only increases by a factor of 1.5. The relaxation of the film lattice temperature is well-described by one-dimensional thermal transport to the substrate^31,35,36^. At tens of ns, the relaxation can be modeled by an exponential decay plus an offset. A careful evaluation of the time-dependent film temperature shows that the lattice cooling at longer time scales cannot explain the observed slowing down of CO recovery (Supplementary Note 2).

To quantify the dynamical scaling of slowing down, we plot the recovery time constants of the CO and lattice diffraction peaks as a function of the film temperatures in Fig. 3e. Approaching *T*_c_, τ_CO_ increases by two orders of magnitude, while τ_lattice_ remains below 7 ns across the CO phase transition, which clearly indicates the recovery of the CO phase does not follow the recovery of the lattice. Under similar pump laser fluence, the concurrent slowing down of the recovery of both the optical reflectivity and the CO superlattice peak around 190 K suggests that the dielectric constant at 1.1 μm is related to the charge ordering, although the probe photon energy of 1.13 eV is much higher than the CO-induced energy gap of 0.13 eV^32^. This observation indicates the macroscopic optical properties across a wide infrared spectrum range in LSFO are highly correlated with the formation of charge ordering on longer-than-thermal recovery time scales. Measurements at other pump fluences are consistent with this observation (Supplementary Note 3). The time constant of the slowing down as a function of temperature is fit to a power law *τ* = *τ*_0_ (1−*T*/*T*_c_)^−*Δ*^ where *T*_c_ *=* 200 K, *Δ* is the scaling exponent, and *τ*_0_ is a constant^25^. The best fit yields *Δ* = 1.25 ± 0.10, shown by the magenta curve in Fig. 3e. The time constant of optical reflectivity recovery is fit by the same function and shown as the blue curve in Fig. 2b, with *Δ* = 1.06 ± 0.16. In conventional second-order critical phenomena, the measured scaling exponent *Δ* is equivalent to *vz* (*v* and *z* are two critical indices^37^) and approximately agrees with 1.3 and 1.37 for three-dimensional (3D) Ising^38^ and Heisenberg^39^ models, respectively, and differs from 2.16 for a two-dimensional model^40,41^. However, we point out that the phase transition in LSFO is first-order-like, because a latent heat is present at the transition in bulk samples^42^. In addition, our electrical transport measurements (Fig. 1b) and the antiferromagnetic order parameter reported previously^29^ are weakly hysteretic as a function of temperature. Thus, our measurements show a pseudo-critical phenomenon near a weakly first-order phase transition^25^, rather than conventional second-order critical phenomena^37^. We also note that the attempted fit for the nucleation and growth model does not agree with the scaling of the time constant as a function of temperatures (Supplementary Note 4). Therefore, the observed scaling cannot be explained by the reduction of nucleation rate of CO domains as system temperature approaches *T*_c_, consistent with no discernible changes of coherence length of CO domains upon optical excitation.

### First-principles calculations

**Fig. 4:**
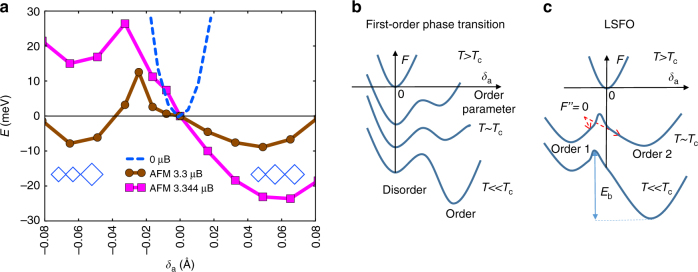
Results of DFT + *U* calculations. **a** The total energy curve calculated using DFT + *U* as a function of the Fe–O bond length difference *δ*_a_ (see inset Fig. 1b) along [111] axis at various Bohr magnetons (μ_B_). The curves with 3.3 and 3.344 μ_B_ was obtained by *U* = 3.7 eV and 3.8 eV in DFT + *U* while fixing the *U* / *J* ratio. The arrays of blue diamonds represent the ordering of the small and large oxygen octahedrons around *δ*_a_ = ± 0.06 Å. **b** The schematic energy potential surfaces for the typical first-order phase transition at various temperatures. **c** The energy potential surface for LSFO shows the degenerate charge ordered states (order 1 and order 2) around the transition temperature. *E*_b_ is the energy barrier between two competing charge-ordered states.  *F*'' is the second derivative of total energy

To further understand the origin of the slowing down, we calculated the total energy of LSFO using the first principles DFT + *U* method, which illustrates the recovery pathways of coupled degrees of freedom during a first-order phase transition. First, we performed the structural relaxation using *U* = 5 eV and *J* = 1 eV. We found that the ground state of LSFO is charge ordered with an associated structural distortion as a result of antiferromagnetic ordering, consistent with the previous DFT + *U* calculations^43^. The resulting structural distortion is characterized by an average Fe–O bond-length difference *δ*_a_ = 0.06 Å (inset, Fig. 1b) between different Fe sites, with an average AFM moment of 3.7 μ_B_. Since DFT + *U* is a zero-temperature theory, we simulate the effect of temperature by varying the interaction parameters (*U* and *J*) to tune the resulting magnetic moment, which is controlled by the sample temperature experimentally. Without any magnetic interactions, i.e., *U* = *J* = 0, corresponding to a sample temperature is above *T*_c_, the energy surface shows no CO or accompanying structural distortion. The calculation thus agrees with a magnetization-driven CO in LSFO^27^. The structural pathway as a function of order parameter δ_a_ is determined by interpolating the CO structure relaxed using *U* = 5 eV and *J* = 1 eV and the non-CO structure relaxed using *U* = *J* = 0 eV. We then vary *U* values from *U* = 5 eV for the low-temperature CO state to *U* = 0 eV for the high temperature state without CO, while fixing the *U / J* ratio to 5. This is used to study the qualitative features of the energy landscapes between two local minima by simulating an increase of sample temperature. The structural pathway is fixed, while we explore the energy landscapes qualitatively due to the change in temperature by varying *U*. While the resulting magnetic moments and energy landscapes can be changed by tuning *U*, the calculated moments for reasonable *U* (3−5 eV) are on the order of 3 μ_B_, comparable with the experimental values^44,45^. At *T* « *T*_c_, our calculations show that the large AFM spin exchange energy between Fe^3+^ ions dominates the energetics and gives rise to one minimum energy state at *δ*_a_ *=* 0.06 Å, accounting for Fe^5+^–Fe^3+^–Fe^3+^ order or small-large-large oxygen octahedra, as illustrated by diamonds in Fig. 4a. As the AFM moment was reduced, simulating an increase in sample temperature, we discovered that a metastable state starts to emerge at *δ*_a_ = −0.06 Å, with Fe^4+^–Fe^4+^–Fe^3+^ ordering or small-small-large oxygen octahedra. By reducing the value of *U* to 3.7 eV, which corresponds to a magnetic moment of 3.3 μ_B_ per Fe, the energy of this metastable state at *δ*_a_ = −0.06 Å becomes degenerate with the state at *δ*_a_ = 0.06 Å, giving rise to the coexistence of two competing CO states with nonzero energy barrier. While the magnetic moments and relative energies of two CO states may sensitively depend on the value of *U*, the position of the two CO states, i.e., the values of *δ*_a_ at two local energy minima, are not sensitive to *U*.

## Discussion

The DFT + *U* calculation shows the energetics of the system is driven by magnetic exchange. Comparing with a typical first-order (Fig. 4b) or second-order phase transition, the energy surfaces exhibit unique characteristics that govern the electronic dynamics. First, the energy barrier *E*_b_ is small, but persists with non-zero AFM moments, which is consistent with the signature of a weakly first-order instead of a typical second-order phase transition. Second, the existence of two nearly degenerate energy states and the rapid quench following the heat pulse provides the opportunity for the system to enter the regime of spinodal decomposition^46^. In this scenario, following the initial laser excitation, the system is ‘quenched’ with *δ*_a_ = 0 (disordered state) from temperatures above *T*_c_ due to relatively fast cooling of the film lattice temperature on the ns timescale. At this point, the system is in the unstable ‘spinodal’ regime where the second derivative of the free energy is less than zero. There is no energy barrier to the formation of the CO states, and the kinetics is limited only by diffusion^46^. Furthermore, this process slows down in the vicinity of spinodal points where the second derivative of the free energy is zero (Fig. 4c) and the diffusion constant becomes vanishingly small^25^. During this process, the domain size does not change, consistent with our observation. At later stages, beyond our measurement time window, processes similar to Ostwald ripening can occur that will increase the coherence length of CO domains^46^. A direct observation of the associated kinetics of spinodal decomposition needs time-resolved resonant X-ray diffraction microscopy with sufficient spatiotemporal resolution which is beyond the scope of this work. At low temperatures (*T* « *T*_c_), as schematically shown in (Fig. 4c), the deep potential well is associated with a strong restoring force that gives rise to a fast recovery from the excited state following a well-defined recovery pathway.

In summary, we observed that the recovery of an electronic phase slows down, becoming longer than the thermally driven processes in a photo-excited LSFO thin film. The multimodal probes via transient optical reflectivity and CO superlattice diffraction allow direct correlation between optical properties and the long-range electronic ordering in the time domain. The electronic recovery is significantly different from the relaxation of the average lattice parameter, providing decisive evidence of the recovery of the electronic phase that is not determined by lattice cooling. First principles DFT + *U* calculations elucidate a microscopic picture of magnetic-interaction driven slowing down and suggest a pseudo-critical phenomenon close to a weakly first-order phase transition. Our combined experimental and theoretical investigation provides experimental verification and mechanistic insight on an unconventional critical behavior and the interplay of multiple degrees of freedom on unusually long time scales at an electronic phase transition.

## Methods

### Sample preparation and characterization

Epitaxial La_1/3_Sr_2/3_FeO_3_ (LSFO) thin films were grown using ozone-assisted molecular beam epitaxy (MBE) on (111)-oriented SrTiO_3_ (STO) substrates. Prior to growth, trichloroethylene was used to remove organic contaminants from the substrate surface. The co-deposited elemental materials La, Sr, and Fe were evaporated from effusion cells under an ozone environment with partial pressure of 3 × 10^−6^ mbar, with the substrate temperature maintained at 680 °C. The evaporation rates were determined for each material from a quartz crystal thickness monitor that was calibrated to within 2% from Rutherford backscattering measurements. The film thickness and surface symmetry were monitored in real time from reflection high-energy electron diffraction (RHEED) intensity oscillations. Brief anneal periods of ~30 s followed the completion of each unit cell layer. After the LSFO deposition, the samples were cooled down to room temperature in an environment of 3 × 10^−6^ mbar of O_3_. The 70-nm thick LSFO sample was characterized by static X-ray diffraction and the results are shown in Supplementary Fig. 2. The integrated intensity of the CO peak was measured as a function of the sample temperature, showing the CO emerges at 200 K, and four-point probe measurement of the sheet resistance shows a hysteresis loop at the transition temperature, shown in Fig. 1b.

### Experimental setup

The ultrafast optical pump-probe experiment is shown schematically in Supplementary Fig. 1a. An optical parametric amplifier (OPA) was pumped by a femtosecond Ti:Al_2_O_3_ laser at 1 kHz repetition rate. The output wavelength of the OPA was doubled to *λ* = 420 nm to excite the LSFO sample, with a pulse duration of 100 fs. A Nd:YAG laser was electronically synchronized with the femtosecond laser to perform asynchronous optical sampling measurements. The output of the YAG laser with a pulse duration of ~100 fs at the wavelength of 1064 nm was focused into a sapphire plate to generate white light with wavelength from 900 nm to 1300 nm. The probing white light was re-focused to be smaller than and spatially overlapped with the pump laser beam on the LSFO sample surface. The reflected white light was analyzed by a spectrometer. The sample temperature was controlled from 78 K to room temperature. In the time-resolved hard X-ray diffraction experiment shown in Supplementary Fig. 1b, the pump laser pulse was derived from the third harmonic generation (THG) of a high repetition rate (54 kHz) Nd:YAG laser, with 355 nm central wavelength and ~10 ps pulse duration. Use of a high-repetition-rate laser is essential to achieve high signal-to-noise ratio for probing time-resolved CO X-ray diffraction. X-ray pulses at 12 keV photon energy and ~100 ps pulse duration were focused by Kirkpatrick-Baez mirrors to a beam size of ~50 µm, smaller than the focused pump laser beam size of ~220 µm. An area detector (Pilatus100K, DECTRIS Ltd.) gated at 54 kHz was used to detect the diffraction intensity. The LSFO sample was mounted on a six-circle diffractometer (Huber GmbH.) in a cryostat with temperature control from 30 K to 300 K.

### DFT calculations

The DFT + *U* calculations were performed using the Vienna ab-initio simulation package (VASP). We adopted the generalized gradient approximation (GGA) exchange-correlation functional for simulations using an energy cutoff of 600 eV and a *k*-point mesh of 8×8×2 aligning the *z*-axis along the [111] direction. We use the supercell of LSFO to accommodate both the antiferromagnetic spin configuration and the octahedral expansion or collapse. The supercell elongated along the [111] direction contains 2 La atoms, 4 Sr atoms, 6 Fe atoms, and 18 O atoms allowing the expansion or the collapse of 6 FeO_6_ octahedral volumes. The convergence of the total energy calculation was reached if the consecutive energy difference was within 10^−4^ eV and the atomic forces of all ions were required to be smaller than 0.01 eV/Å for ionic relaxations. The “+*U*” interaction Hamiltonian was adopted using the rotationally invariant form and the DFT Hamiltonian part did not include the spin-exchange splitting (i.e., the DFT energy part was a function of spin un-polarized charge density, and the spin exchange interaction is entirely treated within the correlated Fe *d* orbitals) aligning with the spirit of DFT + dynamical mean field theory, as implemented in VASP using the option of “LDAUTYPE = 4”. The widely used spin-DFT + *U* implementation, i.e., the spin polarization accounts for both the charge density and the correlated orbitals, often overestimates the spin-exchange interaction in several other systems^47–49^. Especially for this LSFO material, the resulting magnetic moments and energy curves computed using spin-DFT + *U* were insensitive to the change of *U* and *J* parameters since the DFT part alone already accounts for the large portion of the spin-exchange interaction.

### Code availability

The codes for numerical calculations are available from the corresponding author upon reasonable request.

### Data availability

The data that support the findings of this study are available from the corresponding authors upon reasonable request.

## Electronic supplementary material

Supplementary Information
