# Supplementary material for: Unconventional slowing down of electronic recovery in photoexcited charge-ordered La1/3Sr2/3FeO3
Source: Nat Commun. 2018 May 4;9:1799. doi: 10.1038/s41467-018-04199-4 (PMC5935711; doi:10.1038/s41467-018-04199-4)
Supplement: Supplementary file 1 — Supplementary Information [file 41467_2018_4199_MOESM1_ESM.pdf]

Supplementary Information for

Unconventional slowing down of electronic recovery in photoexcited charge-ordered $La_{1/3}Sr_{2/3}FeO_3$

Yi Zhu *et al.*

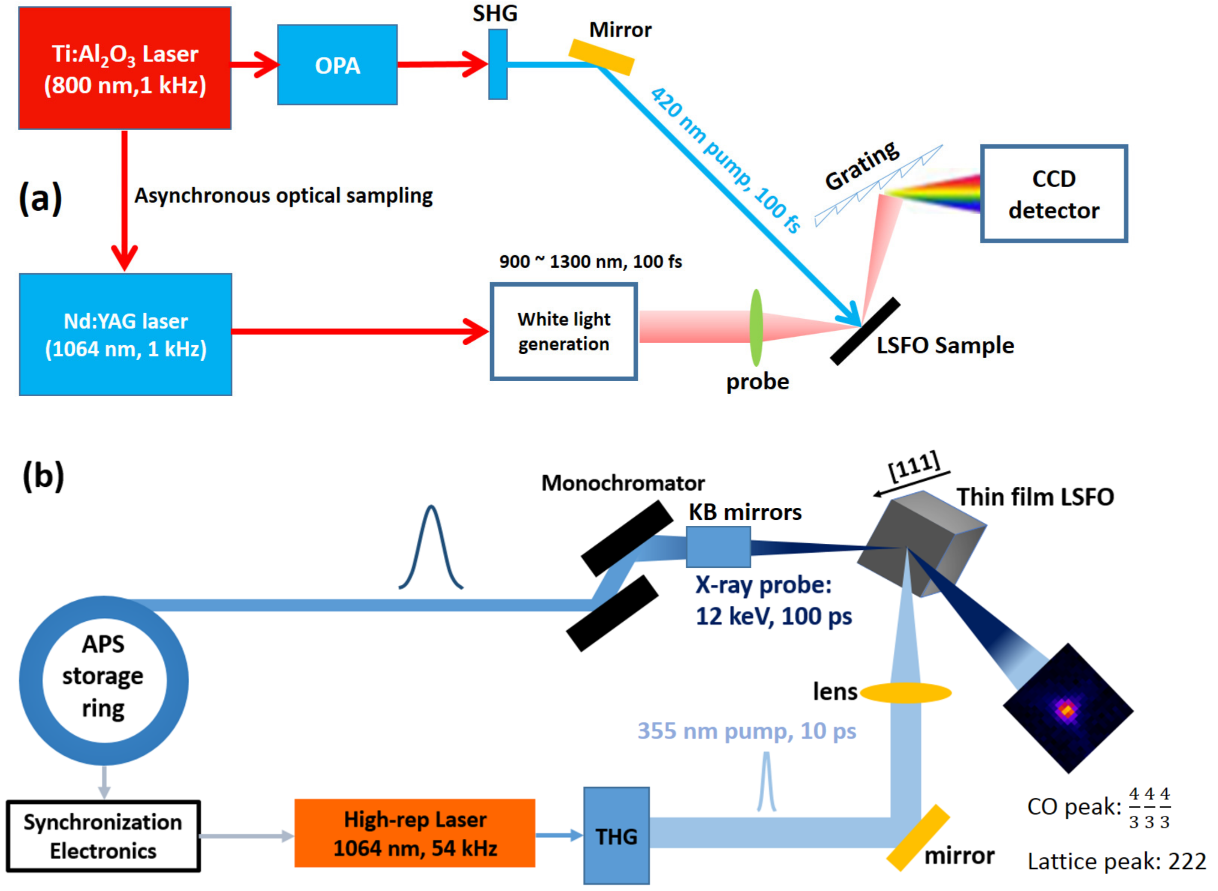

Supplementary Figure 1 Experimental setups. (a) Ultrafast optical pump-probe measurements. OPA: optical parametric amplifier. SHG: second harmonic generation. (b) Time-resolved hard x-ray diffraction measurements. THG: third harmonic generation.

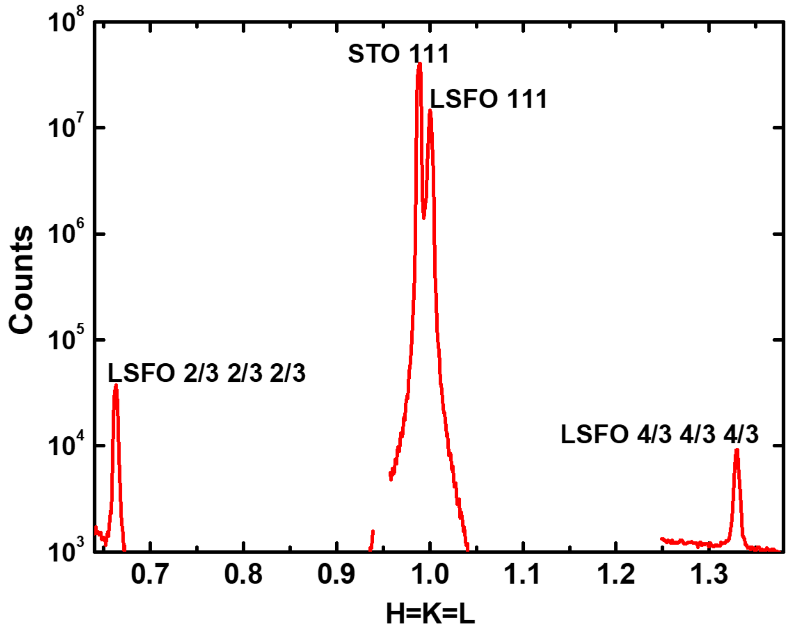

Supplementary Figure 2 Static LSFO thin-film X-ray diffraction. This radial scan along the out-of-plane [111] direction was performed at T=121 K. Miller indices HKL are indexed with respect to the Bragg peak of LSFO film in the ground state.

Supplementary Note 1 Transient optical reflectivity measurements

The slowing down of the recovery of transient optical reflectivity is universal across the probing spectrum close to T_c. This is clearly shown in two representative plots of time- and spectra-dependent reflectivity at 196 K and 199 K, respectively (Supplementary Figure 3). In the main text, we select the optical probe wavelength of 1100 nm to represent the slowing down of reflectivity recovery because the probing spectral intensity close to the fundamental wavelength of 1064 nm gives a high signal-to-noise ratio (SNR).

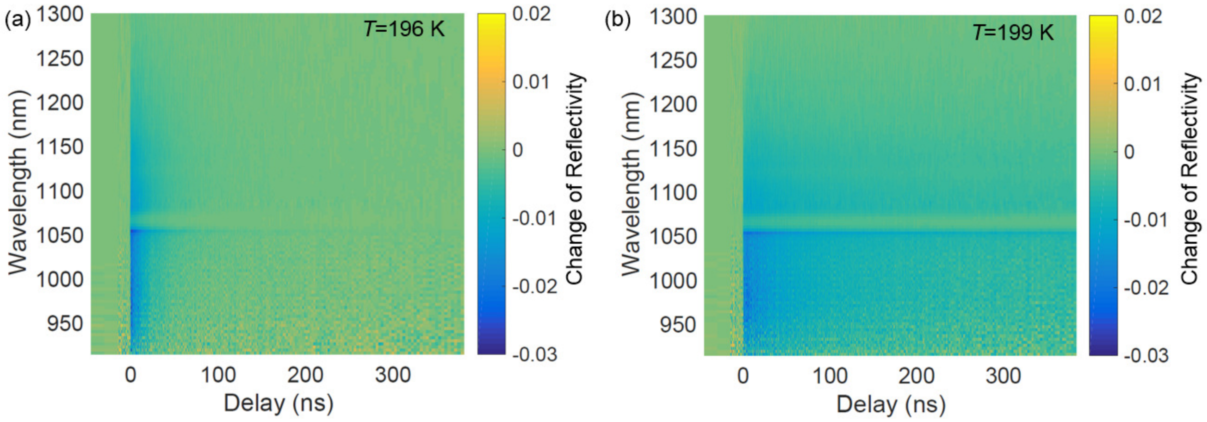

Supplementary Figure 3 The time- and spectra-dependent reflectivity change measured at 196 K (a) and 199 K (b). The spectrum gap between 1050 to 1070 nm is an artifact due to the notch filter for filtering the probe wavelength at 1064 nm used for white-light generation.

Supplementary Note 2 Lattice recovery and the thermal transport process

The representative lattice dynamics measured by the change of 222 Bragg reflection as a function of time at 121 K are shown in Supplementary Figure 4. Similar measurements are performed at various temperatures, and the recovery time of the lattice constant is summarized in Fig. 3e.

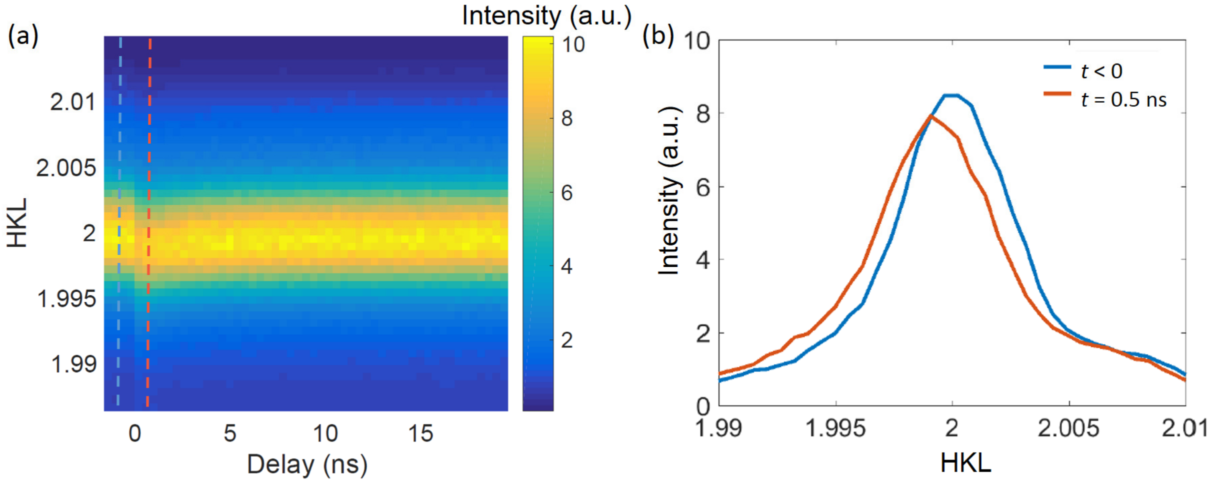

Supplementary Figure 4 Time-dependent lattice Bragg peak. (a) Radial scans of the lattice 222 peak as a function of delay, with an absorbed laser fluence of 2.9 mJ cm^{-2}. (b) The radial scans of the lattice before and after laser excitation as marked by the corresponding color dashed lines in (a).

The maximum temperature jump ΔT is 24 K at 100 ps, calculated based on the shift of the 222 Bragg peak. The Poisson effect needs to be taken into account due to (1) the in-plane constraint of epitaxial strain from the substrate (2) longer time scale of the heat transport along the in-plane direction than that along out-of-plane direction upon optical excitation[1]. The resulting temperature change is $\Delta T = \frac{\varepsilon}{\alpha}(1-v)/(1+v)$, where ε is the observed strain of 0.04% and α is the thermal expansion coefficient approximated by the value of $La_{0.25}Fe_{0.75}FeO_3$ 1.55×10^{-5} K^{-1} [2], and v is Poisson's ratio 0.318, approximated by the value of $LaFeO_3$ [3].

The recovery of the lattice dynamics was measured by monitoring the 222 Bragg peak shift. Measurement 1 and 2 were performed at a sample temperature of 121 K and 300 K respectively, shown in Supplementary Figure 4(a). The agreement of these scans also shows that the lattice cooling is not sensitive to the static sample temperature; this is expected because no structural phase transition is involved in the film, and the thermal conductivity of the substrate ($SrTiO_3$) does not change significantly between 121 and 300 K[4]. Using parameters listed in Supplementary Table 1, we calculate the time-dependent film temperature by solving the thermal transport equation (e.g., see Sec. 4 in Ref. [5], and the Supplemental Material of Ref. [1]). The results are shown as a solid curve in Supplementary Figure 5 and agree with the measurement well.

The measured strain at a delay of 200 ns only corresponds to a film temperature increase of 0.5 K, on the order of the sensitivity of the measurement. At this delay, however, the CO diffraction intensity is still far from a full recovery as shown by the pink curve in Fig. 3d, significantly deviating from the lattice recovery.

Parameters	$La_{1/3}Sr_{2/3}FeO_3$	$SrTiO_3$
Thermal conductivity (W m^{-1} K^{-1})	5 [6]	10.7 [4]
Heat Capacity (J g^{-1} K^{-1})	0.4 [7]	0.57 [8]
Kapitza Constant (W cm^{-2} K^{-1})	10000 [9]	

Supplementary Table 1 Thermal properties of the film and substrate. These parameters were used for the one-dimensional thermal transport calculation.

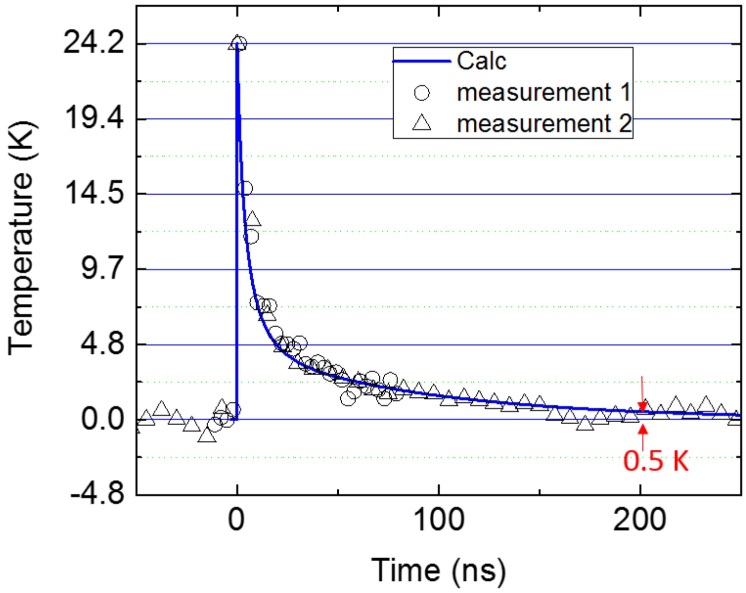

Supplementary Figure 5 Time-dependent film temperature. The calculated (solid line) and measured (open marks) temperature change of the film as a function of time.

Supplementary Note 3 Steady-state sample temperature calibration

Due to high repetition rate of the pump laser (54 kHz) used in the time-resolved x-ray diffraction experiments, the local film temperature under pump laser illumination was higher than the temperature measured by the platinum resistor (PT-100) sensor attached to the sample holder. We calibrated the "steady-state" sample temperature by comparing the CO diffraction intensity with laser illumination to the static temperature-dependent CO intensity measurements, shown in Supplementary Figure 6a. In this regime below the phase transition temperature of 200 K, the sample temperature is proportional to the incident laser power since the involved latent heat is a small fraction of the total latent heat of LSFO [7].

The CO peak intensity measured 1 ns before laser excitation was used to gauge the local film temperature by comparing it with the static temperature-dependent CO intensity. The measurement was performed with the temperature sensor reading T = 178 K at zero laser fluence. The CO diffraction intensity was measured as a function of the laser fluence. By adjusting the scaling of the horizontal axis on the bottom of Supplementary Figure 6a, the change of diffraction intensity

as a function of absorbed fluence (square) was matched to that as a function of temperature (triangle), so that the bottom (fluence) and top (temperature) horizontal axes have a one-to-one mapping. For example, at the absorbed pump laser fluence 2.9 mJ cm^{-2} indicated by the red arrows, the photoinduced CO peak intensity decrease (-16%) before time zero is comparable to that when the sample temperature increases from 178 to 191 K, which means the local sample temperature is 13 K higher than the measured sample holder temperature. The local sample temperature at other pump laser fluencies thus can be calibrated accordingly.

The accuracy of the sample temperature calibration is further confirmed by the slowing down of the recovery dynamics measured under different pump laser fluences. Supplementary Figure 6-b summarizes the recovery time constants as a function of the calibrated temperatures, measured by probing CO diffraction peak and the optical reflectivity. For the optical pump-probe experiment at 1 kHz repetition rate, the average pump laser heating effect is negligible. For time-resolved x-ray diffraction experiments, the corresponding temperature jump at absorbed pump laser fluences of 2.9, 2.3 and 1.5 mJ cm^{-2} are 13 K, 10.3 K, and 6.7 K respectively. After calibrating the film temperature, the slowing down starts to occur around 190 K (Supplementary Figure 6b) regardless of the pump fluences, indicating that the calibration procedure helps measure the local film temperature reliably. We estimate the temperature calibration error is 1 K.

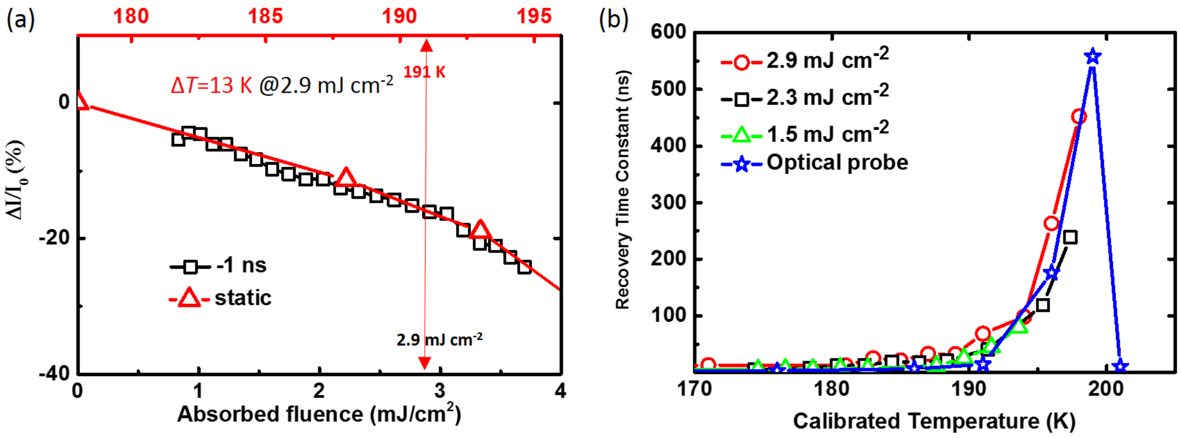

Supplementary Figure 6 Calibration of film temperatures for time-resolved x-ray diffraction measurements. (a) Relative CO peak intensity change as a function of the absorbed pump laser fluence (black) and static film temperature (red). The film temperature measured 1 ns before the laser excitation is 178 K. (b) The CO recovery time constant as a function of temperatures measured at different absorbed pump laser fluences. Optical pump-probe measurements from Fig. 2b are also shown by stars for comparison.

Supplementary Note 4 Fitting procedures

The results of time-dependent x-ray diffraction measurements shown in Fig. 3b and 3d were fit by an exponential decay function: $I(t) = I_0 \exp\left(-\frac{t}{\tau}\right) + c$, where I_0 and c are constants and τ is the decay time constant.

The temperature-dependent recovery time constant of CO diffraction intensity (Fig. 3e) is fit by the scaling law: $\tau = \tau_0\,(1\text{-}T/T_c)^{-\Delta}$. By fixing $T_c = 200$ K as experimentally measured, the best fit yields τ_0=1.5 ± 0.6 ns and Δ = 1.25 ± 0.10. Since the fitting results are sensitive to the film temperature, we studied the impact of the film temperature on the fitting results. Using x-ray data as an example, the fitting results at various T_c are summarized in Supplementary Table 2. Considering the temperature uncertainty of 1 K in the experiment, we show the mean critical exponent Δ=1.24 ± 0.19 with the error bar as the standard error of the mean. The increase of uncertainty of Δ due to uncertainty of sample temperature of 1 K does not affect the conclusions.

Fitting results	T_c (K)		
	199	200	201
Δ (x-ray)	0.90 ± 0.09	1.25 ± 0.10	1.57 ± 0.11
R-square	0.947	0.968	0.977

Supplementary Table 2 The fitting results and fitting errors at various fixed T_c.

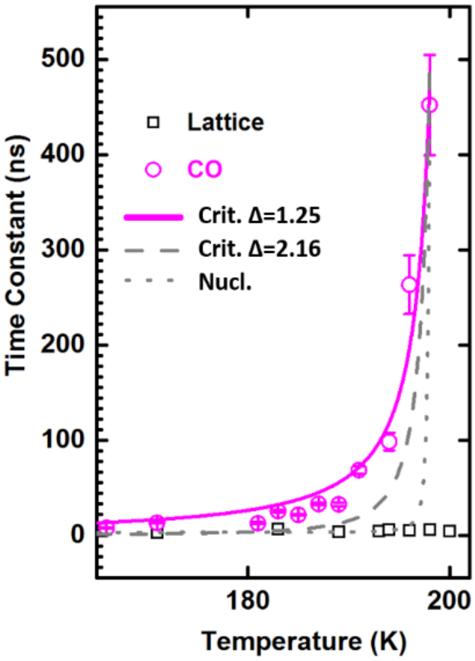

Supplementary Figure 7 Scaling of the slowing down as a function of temperatures. The magenta and dashed gray curves show the fit to critical slowing down model (Crit.) with exponent Δ = 1.25 and 2.16, respectively. The dotted curve shows the fit to the nucleation and growth model (Nucl.). The error bars show the fitting errors of the exponential decay function.

To compare with the critical scaling of the 2D Ising model[10,11], we fixed Δ = 2.16 and T_c=200 K. The best-fit curve deviates from the data points (Supplementary Figure 7). To compare with the nucleation and growth model, the time constant of the recovery of CO diffraction intensity τ is

proportional to the temperature-dependent nucleation rate of CO domains $N = N_0 \exp(-\frac{B}{(T-T_c)^2})$ [12]. Therefore, $\tau = \tau_0 \exp\left(\frac{B}{(T-T_c)^2}\right)$. Since τ_0 ~3 ns as shown in our measurements, we fixed τ_0 =3 ns and T_c = 200 K. The fit yields B=20 K^2 and is shown in the dotted line of Supplementary Figure 7. The deviation of the dotted curve from the measured data points indicates that the temperature-dependent nucleation and growth does not explain the observed slowing down well.

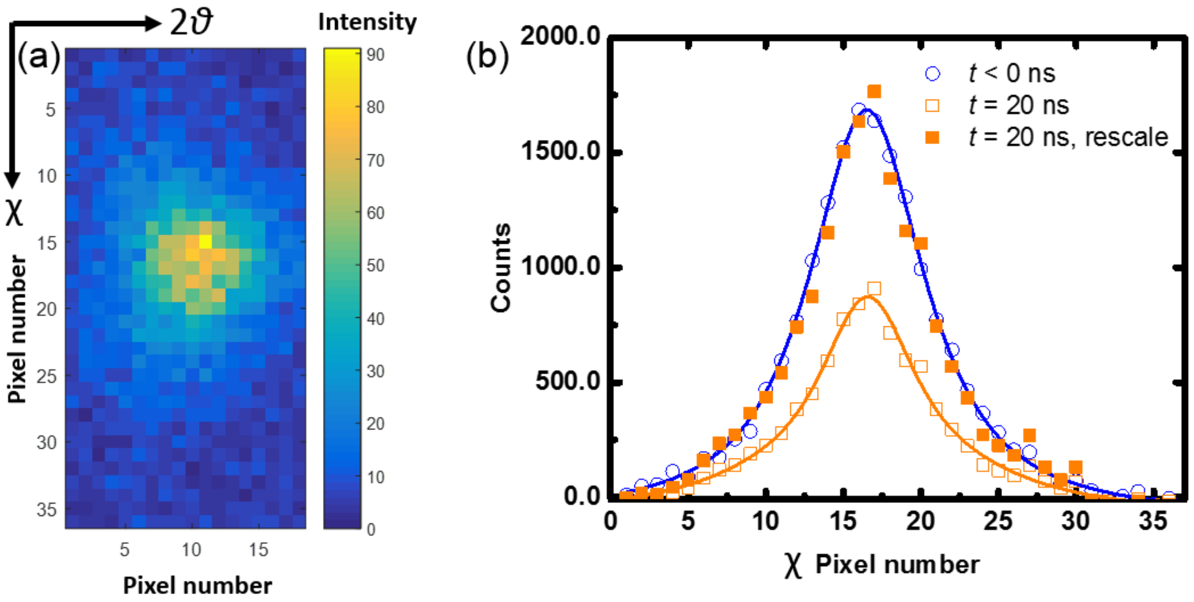

Supplementary Figure 8 In-plane CO Bragg peak width measured at T=196 K. (a) A raw detector image of CO $\frac{4}{3}\frac{4}{3}\frac{4}{3}$ diffraction peak before laser excitation. (b) The CO Bragg peak is integrated along the 2θ direction to show the peak width along χ-direction before and after the laser excitation (open circles and open squares, respectively). The solid squares are rescaled by a factor of 1.94 to directly compare the shapes of the peaks. The solid curves are Voigt fits. No discernible change in peak width is observed.

Supplementary References:

1. Wen, H. *et al.* Electronic Origin of Ultrafast Photoinduced Strain in $BiFeO_3$. *Physical Review Letters* **110,** 037601 (2013).
2. Fossdal, A. *et al.* Crystal structure and thermal expansion of La1- xSrxFeO3- δ materials. *Journal of the American Ceramic Society* **87,** 1952–1958 (2004).
3. Romero, M., Escamilla, R., Marquina, V. & Gómez, R. Structural and mechanic properties of $RFeO_3$ with R = Y, Eu and La perovskites: a first-principles calculation. *The European Physical Journal D* **69,** 177 (2015).
4. Yu, C., Scullin, M. L., Huijben, M., Ramesh, R. & Majumdar, A. Thermal conductivity reduction in oxygen-deficient strontium titanates. *Applied Physics Letters* **92,** 191911 (2008).
5. Shayduk, R. *et al.* Nanoscale heat transport studied by high-resolution time-resolved x-ray diffraction. *New Journal of Physics* **13,** 093032 (2011).

6. Zhou, J.-S. & Goodenough, J. B. Dynamic Jahn-Teller distortions and thermal conductivity in La 1 − x Sr x MnO 3 crystals. *Physical Review B* **64,** 024421 (2001).
7. Approximate by the value of $LaFeO_3$, Stølen, S., Grønvold, F., Brinks, H., Atake, T. & Mori, H. Heat capacity and thermodynamic properties of $LaFeO_3$ and $LaCoO_3$ from T= 13 K to T= 1000 K. *The Journal of Chemical Thermodynamics* **30,** 365–377 (1998).
8. Amirov, A. A. *et al.* Specific features of the thermal, magnetic, and dielectric properties of multiferroics BiFeO3 and $Bi_{0.95}La_{0.05}FeO_3$. *Phys. Solid State* **51,** 1189 (2009).
9. Approximate by the value of oxide interface, Chen, F. *et al.* Ultrafast terahertz-field-driven ionic response in ferroelectric $BaTiO_3$. *Physical Review B* **94,** 180104(R) (2016).
10. Nightingale, M. P. & Blöte, H. W. J. Dynamic exponent of the two-dimensional ising model and monte carlo computation of the subdominant eigenvalue of the stochastic matrix. *Physical Review Letters* **76,** 4548 (1996).
11. Dunlavy, M. J. & Venus, D. Critical slowing down in the two-dimensional Ising model measured using ferromagnetic ultrathin films. *Physical Review B* **71,** 144406 (2005).
12. Porter, D. A., Easterling, K. E. & Sherif, M. Y. *Phase Transformations in Metals and Alloys*. (CRC Press, 2009).
